# Assessing Molecular Docking Tools to Guide the Design of Polymeric Materials Formulations: A Case Study of Canola and Soybean Protein

**DOI:** 10.3390/polym14173690

**Published:** 2022-09-05

**Authors:** Frage Abookleesh, Farag E. S. Mosa, Khaled Barakat, Aman Ullah

**Affiliations:** 1 Department of Agricultural, Food and Nutritional Science, University of Alberta, Edmonton, AB T6G 2P5, Canada; 2 Faculty of Pharmacy & Pharmaceutical Sciences, University of Alberta, Edmonton, AB T6G 2H5, Canada

**Keywords:** plasticizers, cross-linkers, blending, plant protein, biopolymer, AutoDock Vina, molecular docking, binding energy, MM-GBSA

## Abstract

After more than 40 years of biopolymer development, the current research is still based on conventional laboratory techniques, which require a large number of experiments. Therefore, finding new research methods are required to accelerate and power the future of biopolymeric development. In this study, promising biopolymer–additive ranking was described using an integrated computer-aided molecular design platform. In this perspective, a set of 21 different additives with plant canola and soy proteins were initially examined by predicting the molecular interactions scores and mode of molecule interactions within the binding site using AutoDock Vina, Molecular Operating Environment (MOE), and Molecular Mechanics/Generalized Born Surface Area (MM-GBSA). The findings of the investigated additives highlighted differences in their binding energy, binding sites, pockets, types, and distance of bonds formed that play crucial roles in protein–additive interactions. Therefore, the molecular docking approach can be used to rank the optimal additive among a set of candidates by predicting their binding affinities. Furthermore, specific molecular-level insights behind protein–additives interactions were provided to explain the ranking results. The highlighted results can provide a set of guidelines for the design of high-performance polymeric materials at the molecular level. As a result, we suggest that the implementation of molecular modeling can serve as a fast and straightforward tool in protein-based bioplastics design, where the correct ranking of additives among sets of candidates is often emphasized. Moreover, these approaches may open new ways for the discovery of new additives and serve as a starting point for more in-depth investigations into this area.

## 1. Introduction

Recently, millions of tons of non-degradable polymers have been produced annually worldwide. It was estimated that plastic consumption increased from 1.7 million metric tons (Mt) in 1950 to 367 Mt in 2020 and accounts for the continued growth in production year after year [1]. Plastics have become essential components of various products and play a significant role in almost all aspects of daily life. This is because of the large-scale production with fewer input costs and favorable features such as tensile and tear strength, barrier properties, and the capability of a heat seal. However, plastic is made from petroleum-based raw materials that are not readily biodegradable. Therefore, it poses serious health and environmental concerns [2] because of its non-degradable nature and persistence in the environment several decades after use [3]. Considering all these issues, the ban on single-use plastics is expanding worldwide, and Canada recently announced a ban on some single-use plastic items [4].

Currently, high-performance polymeric materials have become a more attractive choice for a wide range of demanding applications. In this vein, extensive research efforts have been devoted to developing high-performance polymeric materials to replace their petroleum-based counterparts. Various bio-based polymers have been investigated for the development of biodegradable materials. More specifically, sustainable bio-based polymer alternatives, including polysaccharides, proteins, and lipids [5,6,7,8], have been explored as candidates for bio-based polymeric materials. Among the various types of natural polymers, proteins have been considered the ideal bio-based material for the future development of bio-plastics due to their attractive combination of price, abundance, renewability, and biodegradability. Canola and soy proteins are among the most investigated proteins in biopolymer research. The main components of canola protein are napin (2S albumin) and cruciferin (12S globulin), which are storage proteins. They account for 20% and 60% of the total protein content [9]. Soy protein is composed of a mixture of globular proteins containing 2S, 7S, 11S, and 15S fractions [10]. Canola and soy protein-based polymers can be used as biodegradable films in packaging materials, adhesives, and or even as scaffolds for tissue engineering [11]. These polymers show favorable material properties such as biodegradability, biocompatibility, and lower toxicity than conventional synthetic polymers. Unfortunately, most biopolymer-based materials suffer mechanical problems under low values of applied stress and thermal stability in comparison to synthetic materials [12,13,14]. Therefore, it has always been critical to develop high-performance polymeric materials with exceptional mechanical strength and toughness, thermal stability and, even healable properties for meeting their performance requirements in industry. The creation of plasticized, cross-linked polymers and blended mixtures has been regarded as a promising approach for developing strong and thermally stable polymers [15,16,17]. However, the proprieties of these (bio)polymers can be optimized and improved according to their interactions with the additives used in terms of compatibility, molecular weight, chemical structure, functional groups, and number and positions of active groups. Therefore, the type and the concentration of the additives used strongly affect the bioplastics formation and its final properties [14,18,19]. The selection and utilization of plasticizer, cross-linkers and polymers is a fundamental step in providing biopolymeric materials with satisfactory properties. Selecting a suitable formulation is mainly dependent on the compatibility between the agent and the protein in terms of solubility, molecular weight, polarity, hydrogen bonding, and stability in the formed film, as well as on the efficiency in terms of the ratio [14,20,21]. Hence, it is critical to emphasize that the type of additive differs from one protein to another. Therefore, selection and optimization of the suitable agent prior to being used in the bioplastics film formation is highly required. Several advanced techniques, such as calorimetry, gravimetry, dynamic mechanical analysis, and tensile testing, were used to investigate the modification behavior and thermal and mechanical properties of the modified protein-based films [22].

Computational protein–ligand docking analysis has been one of the most important and basic tools in drug discovery and development. Generally, the process of computational protein–ligand docking begins with a protein target of known crystallographic structure [23,24]. A molecular docking tool is usually used to model the interactions between a protein and small molecules (ligands) at the atomic level by predicting their optimal conformations and expected binding free energies [25,26]. The predicted binding affinity is of great value. It is often used to predict the interactions between two molecules. Thus, protein−ligand docking can provide important insights into the effects of a chemical structure and the chemical nature of noncovalent interactions formed between proteins and ligands [27]. The computational docking methodology is considered a rational tool for drug discovery, and it enables the rapid identification and screening of millions of small molecule drug candidates in an affordable time. It can also be used to inform subsequent validation of conventional in vivo and in vitro models, thus reducing research time and the costs of drug discovery efforts [25,28]. In this context, the experimental selection of biobased additives to promote the formation of bioplastics is not only costly but also time-consuming and generally labor-intensive. Therefore, due to the above-mentioned limitations of conventional approaches, limited efforts have been made to understand protein–additive interactions at the molecular level. Nevertheless, computer-based methods might be an attractive way to better understand how different modifying agents may interact and improve the protein-based bioplastics properties and then help in designing bio-based polymer–additive complexes with superior properties in a smaller number of experiments, lesser time, and lower cost. However, limited efforts have been made to use molecular docking for predicting protein–additive interaction and ranking additive suitability for making bioplastics. Therefore, we hypothesized that molecular docking might be a rapid and reliable method in predicting protein–additive interactions leading to the selection of suitable additives for designing bioplastics with better properties.

Based on the conclusions drawn from the current research in biopolymer formulation, selecting a suitable additive is a key step and can have a strong impact on mechanical, physical, and thermal properties and consequently on the efficacy and application of the formulated biopolymer, as such better properties directly related to a specific interaction between a polymer and particular additive. The latter is motivated by the proposed use of computational studies. In essence, our studies in this area are based on the relationship that exists between the compatibility of protein and particular additive and required improvements according to the action of the additive used. Hence, the better the compatibility between two molecules, the stronger the interaction (affinity between them), and the better the properties. Therefore, the aim of this work was to examine the feasibility of the molecular docking approach as a fast tool to rank a set of potential additives based on their binding score. A total of twenty-one additives of three modification classes (plasticization, cross-linking, blending) were used to investigate their interaction with two plant proteins (canola and soy proteins). We mainly focused on analyzing the binding interactions between proteins and additives to help us understand and address key questions associated with the diversity of protein–additive interactions in terms of binding affinities and specificity among the investigated additives. We, therefore, envisage that the results of our computational study will find widespread use in areas of protein-based bioplastics preparation.

## 2. Materials and Methods

### 2.1. Ligand and Protein Structures

**Figure 1:**
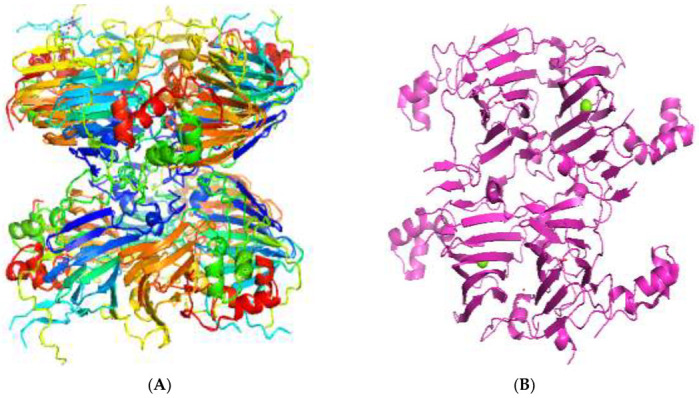
Crystal structure of canola procruciferin, 11S globulin (**A**) and soy glycinin A3B4 subunit (**B**).

The 3D structures of canola procruciferin, 11S globulin (PDB ID: 3KGL), soybean 11S Globulin: Glycinin A3B4 glycinin (PDB ID: 1OD5) were used in this study and downloaded from the Protein Data Bank (PDB). The 3D graphic structures of proteins used in this study are displayed in Figure 1. Twenty-one structurally diverse additives such as plasticizers, cross-linkers, and blinding agents were studied, which are treated as ligands in the docking setup. The 2- and 3D structures of ligands were downloaded from the PubChem Compound Database (National Center for Biotechnology Information; https://pubchem.ncbi.nlm.nih.gov/) (accessed on 5 February 2022). The structures of each additive can be found in Table 1.

### 2.2. Software and Programs Used

AutoDock Vina software employs the Lamarckian genetic algorithm was employed to create input files for our docking calculations [29]. The MOE software with the newly developed GBVI/WSAdG scoring function is used in the study [30]. For each protein structure, the binding sites were predicted using the binding site finder of MOE tool and CASTp analysis [31]. Vina output results were visualized using PyMol [32], Discovery Studio Biovia 2015 [33], and Molecular Visualization VMD [34]. Open babel was used to generate the 3D conformation of the ligand from the SDF format [35]. MM-GBSA module in Schrödinger software was used to study the interactions for each complex resulting from the docking simulations under default parameters [36]. The receiver operating characteristic curve (ROC) and AUC (Area Under the Curve) curves were generated for AutoDuck Vina methodology using OriginLab 2022b version [37].

### 2.3. Preparation and Docking Protocol

Docking was carried out using AutoDock Vina and MOE methodology. Discovery Studio Biovia software was used to prepare the required files by removing water molecules, adding polar hydrogen atoms, and Kollman charges to the protein structures. Then PDBQT files were generated to be used for molecular docking process. For docking protocol, both protein coordinate files were deposited as the input receptors, while each additive was treated as the input ligand. Additives were docked to each target receptor with grid boxes of certain sizes for each receptor according to the pocket size and the number of residues present in each respective binding site. The grid size and the grid center were designated at x, y, and z dimensions. The binding affinities were typically calculated for the selected poses from molecular docking using each software. The free energy for each selected complex was estimated using the prime MM-GBSA energy function in the Schrodinger software package. The complex binding pose with the best score from the MM-GBSA calculation was subject to the mode of binding analysis. Subsequently, after all dockings were performed, the selected complexes were visualized using PyMOL, Discovery Studio, and VMD to display the sizes and locations of binding sites, as well as types and distances of noncovalent interaction. For the visualization section, we report all the hydrogen bonds with a cut of 4 Å and 65 angle cut-off from the position of the docked ligand.

## 3. Results and Discussion

Native protein biopolymer often has a weak performance, and, therefore, processing of biopolymers requires the addition of one or more different additives to obtain the required properties for a particular application. Different types of additives were used to improve the processability and performance of biopolymers. Generally, these improvements are mainly attributed to the amino acid composition, and the diversity of side chains groups of proteins, such as hydroxyl, amino, and carboxyl groups, enables proteins to have multiple reaction options with a variety of additives [38]. As a result, certain important properties can be altered through chemical modification reactions between additives and functional groups, such as hydrophilic or hydrophobic characteristics, elasticity, adsorption, thermal properties, and mechanical resistance [15]. In cross-linking modification process, the biopolymer can be cross-linked through covalent bonds, hydrogen bonds, or Van der Waals bonds cross-linking, depending on the type of the protein cross-linking agent interaction [39]. Recently, several authors have demonstrated the possibility of carrying out the cross-linking process in the modification of the structure of a protein through noncovalent secondary interactions such as hydrogen bonds, electrostatic interactions, and hydrophobic forces, and without unnecessary extra reactions [40,41,42]. However, this cross-linking process may produce structural changes that not only influence the mechanical and microstructural properties of the material but could also make modifications to the functional and application properties [43,44]. On the other hand, plasticizers are the most widely used additives in both synthetic and bioplastics, which are added to improve several properties, such as chain flexibility. Polar plasticizers such as polyols could easily fit into the protein chains and bind at the respective binding sides. Protein–plasticizer interaction could establish a greater number of hydrogen bonds with reactive groups of proteins and cause increases in the free volume. Thereby, the protein biopolymer mobility and material elasticity are increased [42]. However, selecting the proper additives for an application has always been a daunting task. This is because the compatibility and performance of additives vary with the types of protein biopolymers. Multiple parameters can indicate this variation, including molecular weight, polarity, hydrogen bonding (or H-bond), and solubility [45]. For better performance, it is very important to estimate the efficiency or trend of the additives from the readily available parameters that characterize the performance of the intended additive. Unfortunately, no single parameter can be used as an indicator for the compatibility between the polymer and suitable additive. Another challenge in the development of bioplastic materials is that the enhancement of one property is frequently accomplished at the expense of another. Thus, the designing of bioplastic materials demands an intensive assessment of multiple performance criteria in a coordinated manner. An overall and deeper understanding of the connection between the additive and host polymer is highly desirable and might help to overcome the challenges of obtaining a bioplastic with an outstanding property. Therefore, predicting accurate protein–additive binding affinities and exploring the interaction mechanisms among a set of additives can be a valuable tool not only for ranking additive efficiency but can provide insight into the mechanism behind the ranking. However, we do not pretend to be comprehensive in this study, but an overview of the molecular mechanisms behind the protein–additive interaction will be provided. Indeed, it is not possible to uncover the molecular mechanisms behind all observations in a single study, especially in complex polymer systems. Therefore, for every additive class reported in this study, variations in binding scores among a set of every class are reported, and the 3D structures of protein–ligand interactions are analyzed to provide valuable insights for polymeric materials formulations. Moreover, to provide a full picture, insights from our modeling results were combined with a relevant previous experimental study that investigated the same group of additives and proteins.

In the present study, the crystal structure of canola procruciferin, 11S globulin from Brassica napusis, and soybean 11S Globulin (Figure 1) were used as the target proteins, with different classes of biopolymer additives used as ligands. The study aimed to predict and compare the binding affinities that describe the strength of interactions between additives and proteins and then rank the interactions according to their binding energies. The first investigated class of bioplastic additives is plasticizers. In the utilization of biopolymers for bioplastic synthesis, plasticizers are among the most widely used additives. They are typically used as a flexibilizing agent to reduce the glass transition temperature, thus producing a flexible polymer. Seven of the most widely used plasticizers in bioplastic formulations were selected, and their interactions were investigated. The results showed that all the plasticizers docked with negative binding energy values indicative of the affinity of the additives for their target proteins. For most docking protocols, the lowest binding affinity of top-ranked poses is generally regarded as the standard selection in a typical docking analysis. The calculated binding energies of plasticizers’ interactions are shown in Table 2. Comparing the docked plasticizers showed considerable differences in their interaction strength and binding sites with the studied proteins. The binding affinities values for all the plasticizers resulting from the AutoDock Vina and MOE have close scores, and values range (from −2.0 to −5.9 kcal/mol) as reported in Table 2. Both Vina and MOE scores showed low binding scores, indicative of very weak binding, whereas strong interactions were recorded in MM-GBSA energies. Thus, we base our discussion on MM-GBSA calculations to rank the studied additives. 

However, in molecular computational modeling, the Molecular Mechanics/Generalized Born Surface Area (MM-GBSA) binding free energy is an important and widely used method for binding free energy prediction. This method calculates the binding free energies of molecules by combining molecular mechanics calculations and continuum solvation models. Accordingly, the MM-GBSA binding free energy has been successfully applied to various protein–ligands or protein–protein/peptide complexes. It has also been widely reported that the binding scores calculated using MM-GBSA energies can predict binding affinities with higher accuracy than most of the scoring functions. As shown in Table 2, the predicted MM-GBSA of plasticizers are ordered as follow: phthalate (−30.05 kcal/mol) > sorbitol (−28.86 kcal/mol) > triethanolamine (−27.17 kcal/mol) > glycerol (−23.27 kcal/mol) > ethylene glycol (−18.70 kcal/mol) > urea (−15.44 kcal/mol) > formamide (−14.85 kcal/mol). Phthalate, sorbitol, triethanolamine, and glycerol all have higher free binding energy calculated using MM-GBSA compared to that of formamide, urea and ethylene glycol (<−20 kcal/mol). On the other hand, our docking modeling convincingly predicted the similarity trend between the two proteins. This is because canola and soybean proteins are identical in their amino acid profiles [46]. According to the analysis of the soybean protein–plasticizer complex, the lowest binding affinity and MM-GBSA (Table 2) were found for phthalate, followed by sorbitol and triethanolamine, respectively. The correlation coefficient was 0.9197, which reveals that the predictions from binding scores and MM-GBSA methodologies were efficient and capable of ranking plasticizers with similar and diverse structures. The predictions for soybean proteins were strong, and the binding free energies of the plasticizer–soybean complexes fall within a rather broad range, from −7.67 to −60.87 kcal/mol.

According to the theories and mechanisms of plasticizer action, plasticizing efficiency depends on the chemical properties of molecular polarity, volume, and weight. In reality, the interaction between the plasticizer polar groups with the polar group on the polymer is an intermolecular plasticizing step. From the result presented in Table 2 and Table S1, plasticizers with a higher polar group, such as triethanolamine, sorbitol, and phthalate, showed stronger interaction. In this prospect, we suggest that plasticizers that bind strongly with the host polymer matrix are expected to be more stable, thus giving superior and stronger properties. This is essentially the rationale behind the plasticization theories. These results are in agreement with experimental data available in the literature. For example, canola protein plasticization experiments using different polyols plasticizers, such as sorbitol, glycerol, and polyethylene glycol-400, indicated that the best mechanical properties were recorded with sorbitol followed by glycerol [47]. Furthermore, compared to glycerol, sorbitol was found to show higher and the best mechanical properties than glycerol for the plasticization of soy protein-based bioplastic [48]. The observations of this study were also very consistent with the previous study that involved AutoDock Vina and the experimental study of glycerol and sorbitol-plasticized soybean-based bioplastic. The authors reported that the results of AutoDock Vina helped explain the trends with the experimental results of the mechanical properties for the investigated bio-plastics [49]. Aguilar et al., 2020, investigated the effect of different plasticizers on the thermal, physical, and mechanical properties of soy-based bioplastics. They found that glycerol is more compatible with soy-based bioplastics than ethylene glycol. The same study also estimated the plasticizer’s volatility after 9 days of storage. Their results showed only glycerol remained in the bioplastic, highlighting the volatility of ethylene glycol as a primary aging factor [50]. Based on our docking results, this was not surprising since ethylene glycol was found to have a weaker interaction, thus, less stability when compared to glycerol. According to the basic principle of protein–ligand interaction chemistry, and laws of thermodynamics, the complexes with lower energies are more stable. Therefore, assessing binding scores can be confidently used as a parameter to predict and discriminate the additive stability among a set of candidates.

**Figure 2:**
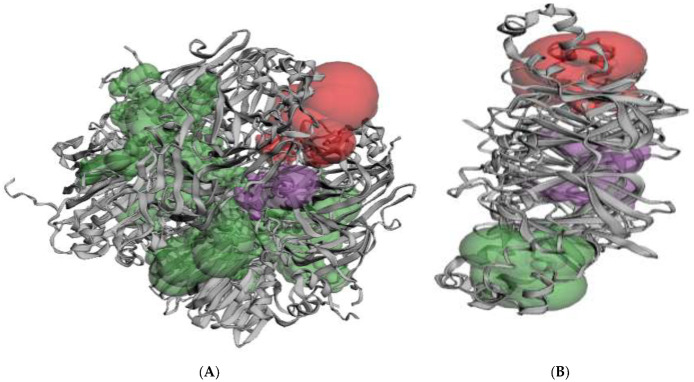
Surface topography of Canola protein (**A**) and Soybean (**B**) (colors represent the three largest pockets).

However, it is clear to note from our results that increasing the molecular weight led to an increase in the strength of the interaction between the protein and plasticizers, regardless of the number of polar groups the plasticizers had or the number of hydrogen bonds formed (Table 1), which are known to promote the protein–plasticizer interactions. Interestingly, none of the covered plasticizers which have a molecular weight lower than 60 g/mol, such as formamide (−14.85 kcal/mol), urea (−15.44 kcal/mol), and ethylene glycol (−18.70 kcal/mol) form a good binding affinity. This observation can be explained by variations in the molecular weight of the plasticizer and ligand binding pockets/cavities, which vary with the protein size. Our observations suggest that the higher the molecular weight of the plasticizers, the better the molecular fit and the stronger the interaction to form a more stable complex. This is due to the large size pocket/cavity of canola protein where larger plasticizers can properly fit and take effects than a smaller plasticizer’s weight. Consequently, a sufficiently high-affinity plasticizer can be confidently expected to be highly specific for its target. Concerning the pocket size, it has been previously reported that protein–ligand binding sites tend to take place within the largest and deepest pocket/cavity on the protein’s surface [51]. In order to assess the role of pocket size, protein structure topology was also investigated using the alpha shape method and CASTp 3.0 method [31]. CASTp pocket measurement is represented as pocket volume based on the solvent-accessible surface (SA) and pocket solvent-accessible surface area (SA). According to our visualization analysis, canola proteins typically have up to 100 pockets/cavities, which vary widely in size. As illustrated in the snapshot (Figure 2A), the size of the largest cavity (Green color) lies within 1523 Alpha Spheres and 11340 (SA) of pocket surface area or 11948 (SA) of pocket volume. This pocket comprises about 276 hydrophobic sites and 676 hydrophilic. Most of the additives investigated in this study were found to bind to this larger pocket/cavity, with the exception of the smaller molecular weight additives, which do not bind to the largest pocket. The size of the second larger cavity (Red) lies within 391 Alpha Spheres and 1270 (SA) or binding site volume of 2677 (SA), comprising around 74 hydrophobic sites and 174 hydrophilic sites. It should be noted that the interactions of the small molecular weight plasticizers such as formamide, urea, and ethylene glycol took place only at the smaller pocket/cavity. Moreover, water is the main solvent used in natural bioplastic fabrication. The co-plasticizing effect of water on proteins and polysaccharides has been widely investigated and demonstrated [52]. Our result showed that water has the lowest interaction with a binding affinity of −2.00 kcal/mol. The observation of water-protein interaction shows that water has a binding site composed of a single small pocket with a surface area of 7.426 (SA) and several small neighboring pockets. Hence, water seems to be a bit lower in efficiency than formamide and urea. Since their favorable interactions are not the largest pockets, therefore they did not show a stronger interaction, and such plasticizers are not expected to have longer stability within the bioplastic formulated. Contrary to canola protein, all plasticizers were docked into a single pocket, which contains the most active sites on the soybean protein. This is due to the smaller pocket/cavity size of soybean protein. The size of the largest pocket/cavity of soybean protein (Figure 2B) lies within 261of Alpha Spheres, and 811 (SA) comprising about 48 hydrophobic sites and 138 hydrophilic sites, whereas the size of the second large cavity (Red) lies within 61 of Alpha Spheres, and 769 (SA) or binding site volume of 1748 (SA). 

Furthermore, the effect of molecular weight of plasticizers from experimental investigations has been the focus of a number of publications. Admittedly, the selection of an ideal molecular weight of plasticizer used in bioplastic formulation is recommended to be fully considered. In addition to its role in the protein–plasticizer interaction, the molecular weight of the additive can play a crucial role in the manufacturing process, such as time, diffusion, and energy required for the absorption process into the host polymer, as well as migration and stability of plasticizer within the polymer. However, there is no clear trend in the literature about the effect of the molecular mass of plasticizers on bioplastic properties, and there are numerous levels of discrepancies among these in the literature. Therefore, in terms of plasticizer efficiency, key conclusions from our docking observations suggest that molecular weight is among the key contributors to the specificity of interactions. This finding motivated us to hypothesize that the role of molecular weight of an additive could be identified based on pocket/cavity size on the host polymer where they bind. Moreover, we are confident that the binding of the plasticizer on the largest pocket/cavity of the polymer plays a crucial role, hence significantly influencing the plasticization efficiency. This implies the necessity to consider the relationship between the pocket/cavity size and plasticizer molecular for the selection of the right choice plasticizer.

In spite of the fact that the values of the binding score are informative of ligand binding in the active pocket, types of molecular interactions between the protein and ligands are indicative of ligand binding in favorable conformations. The wide variety of additive types covered in the study showed different types of interactions, residues, and pocket/cavities. Noncovalent interactions such as hydrogen bonding, electrostatic, π-effects, and hydrophobic effects govern the most attractive interactions between the two molecules. Hydrogen bonds are extremely important, especially in polymer modification interactions which play a key role in the stability and properties of the produced bioplastics.

**Figure 3:**
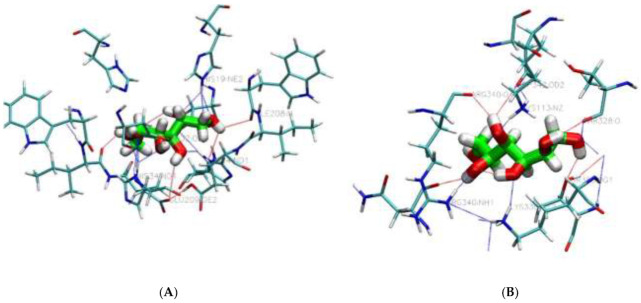
Three-dimensional view of protein–sorbitol interactions of the best poses generated on Canolaprotein (**A**), and Soybean protein (**B**).

To estimate the difference in the noncovalent bond-forming abilities among the investigated plasticizers at the molecular level, visualization of protein–plasticizer interaction, as presented in Figure 3A,B and Table S1, showed that all protein–plasticizer complexes are mainly stabilized by noncovalent interactions, particularly hydrogen, hydrophobic and salt bridges which are mediated by the corresponding amino acid residues in each protein–plasticizer interaction. The results also showed that certain types of polar groups along the plasticizer chains are involved in developing protein–plasticizer hydrogen bonds, replacing the protein–protein interactions in the bioplastics formulation. It has been widely reported that an additive interacts with protein biopolymer through hydrogen bonding, salt bridges, and hydrophobic groups are considered the most suitable candidates for many protein-based polymeric materials. A set of hydrogen bonding interactions with polar side chains, such as tyrosine, threonine, asparagine, serine, cysteine, and glutamine, have been observed at distances within 4 Å. The total number of hydrogen bonds was almost the same for all plasticizers, with the exception of phthalate, which showed lower values but higher hydrophobic interactions, which may play a major role in the process of plasticization. It is known that the formation of hydrogen bonding between the plasticizer and the biopolymer leads to a decrease in intermolecular chain entanglement, which can directly affect the different properties, such as the glass transition (Tg), of polymeric materials. Herein, it is important to emphasize that the formation of extensive hydrogen bonding networks between the plasticizer and host biopolymer might lead to forming a more rigid plasticizer–biopolymer complex and therefore reduce the plasticization efficiency. Thus, the role of hydrogen bonding needs to be assessed through dynamic molecular simulation tools to provide a clearer picture. However, our docking calculations and visualization of 11S globulin of soy protein/plasticization were in accordance with the result obtained by Patnode et al., 2021, in which lysin, serine, threonine, leucine, asparagine, glutamine was the most favorable pose for sorbitol and glycerol plasticizers [50]. Similarly, another study revealed that threonine, lysin, and proline are the key amino acid residues for 11S globulin of soy protein [53]. However, details of all additives interactions within the predicted active pocket of investigated proteins, including key residues involved in the interactions, distance between interacting residues, and additives atom from our modeling, and results found in the literature are presented in Tables S1 and S2.

In general, processing biopolymer without additives is not viable. Therefore, the combination of two or more is a common procedure for the development of high-performance biopolymeric materials. For example, a combination of plasticization with cross-linking or blending with other polymers or the use of all these together is the common procedure for the development of bioplastics. On the other hand, the addition of more than one additive might interface or influence each other and may lead to opposite effects on properties. Therefore, there should be reasons for the polymer-type specific additives, which require a deeper understanding of their interactions at the molecular level, and consequently, these additives must be designed based on the mechanisms of interactions and according to the molecular basis of the components and the biopolymer type. Hence, comprehensive knowledge of the interaction between protein and additives at the molecular level is required and can be directly achieved through the molecular docking approaches in order to effectively guide the continuous discovery and development of high polymeric materials.

Through our investigation in this study, we attempted to gain molecular-level insights into the protein–additive interactions. Therefore, a wide scope of modification classes was investigated throughout this study to help in making any molecular interpretation. Thus, the second class of bioplastic additive investigated in this study was cross-linker. In recent years, the cross-linking approach has been among the most promising research gateway focusing on the development of bioplastics materials. In this approach, a biopolymer can be chemically cross-linked through covalent or noncovalent interactions to form a strong, rigid, three-dimensional network [54]. Contrary to plasticization, the formation of a rigid three-dimensional network of the additive–polymer complex is highly desired and core for cross-linking efficiency. This process can affect some physicochemical properties, including mechanical and thermal performance and enhanced gas barrier properties, among others [55]. Herein, we investigated the specific interaction of different cross-linking agents. We docked seven among the most used cross-linkers to cover different cross-linker reactive groups. Comparison between those cross-linkers allowed detailed discussion about the effects of molecular weight and functional groups, where the effects of additives on molecular weight and functional groups are substantial.

In this section, we are, however, unable to find corresponding experimental results, and therefore, we will limit our discussion to our modeling calculations. From our docking results (Table 3 and Table S1), we observed that all cross-linkers used in this study showed different interaction affinities with the investigated proteins. As shown in Table 3, we observed an increase in the cross-linker interactions in the following order tannic acid > genipin > citric acid > succinic anhydride > maleic anhydride > glutaraldehyde > glyoxal which fell into a range of MM-GBSA from −11.0044 to −60.1099 kcal/mol, with canola proteins. A similar trend was observed with soybean proteins; the tannic acid, followed by genipin, stood out as having the highest interactions, as inferred by the lowest binding energy from the MM-GBSA calculation (Table 3). These results suggested that the stronger interactions of these cross-linkers can stabilize the complex properly and effectively, leading to improvements in some physicochemical properties. However, as indicated by the sign of the correlation coefficient, the binding free energies predicted by MM-GBSA achieved relatively satisfactory correlations with the binding affinity of the AutoDockVina results (correlation coefficient r = 0.9465 and 0.9322 for canola and soybean protein, respectively).

By observing the interaction at the large size pocket/cavity on canola proteins, all cross-linkers properly interacted except glyoxal. Glyoxal (58.04 g/mol) showed the least interaction, and the interactions were found at the second largest pocket/cavity. This result was also indicative of the influence of the additive and pocket/cavity size. The same trend with plasticizer interactions is shown as well. This finding is again consistent with our conclusion, where molecular weight and pocket size on the host polymer play a key role in the interaction, stability, and selection of a suitable additive. Therefore, we thus conclude that the binding location of the additives on the polymer backbone can play a crucial role. This knowledge can be extremely useful in the designing of protein-based bioplastics by understanding and choosing the right additives to overcome the issues of additives competing with each other. The finding led us to seriously consider the effect of two or multiple diverse additive functional groups binding at the same single protein site, either by interacting with largely the same residues or different residues within it. Results from our docking show similarity to the binding site with some of the investigated additives. For example, in the case of canola protein, ARG 190 B is the preferred site for triethanolamine, citric acid, and agar, while HIS 184 B and ARG 190 C were preferred for citric acid and agar. ARG 190 D is the preferred site for sorbitol, genipin, dextran, and cellulose. LEU 181 Fand ASN 186 F are the preferred sites for glycerol and kefiran in which two to three strong hydrogen bonds are formed with each additive. These results indicate that there is lower compatibility between two or more additives such as glycerol with kefiran. Thus, this partly interference may lead to minimizing (within uncertainty) the strength of the interaction. However, comprehensive investigation behind every observation in a single study is not a trivial task, and further investigation of the competition between the intended additives is highly important to obtain the right action for each additive used.

**Figure 4:**
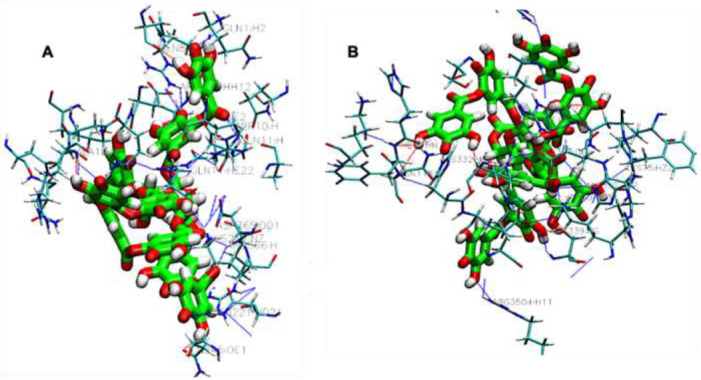
Three-dimensional view of protein–tannic acid interactions of the best poses generated on Canola protein (**A**), and Soybean protein (**B**).

Visualization analysis of the interaction showed that hydrophobic, π-stacking, salt bridges and hydrogen bonds (Table S1) had been found to favor the formation of the complex. Multiple active site residues were involved in the interaction with every cross-linker investigated here. For example, carboxylic acids such as tannic acid and citric acid were shown to actively participate in hydrogen bonding interactions with other polymer networks, improving their properties due to their carboxylic (COO) and hydroxyl (OH) groups. It has been recently reported that the utilization of natural cross-linking agents, such as tannic, acetic acid, and malonic acid, gives the necessary improvements in tensile characteristics and increased stability under aqueous conditions in various biopolymer applications. Our docking results found tannic acid to be the most active compound in this series. The observed MM-GBSA values of tannic acid–canola protein and tannic acid–soybean protein was −52.21 Kcal/mol and −61.10 Kcal/mol, respectively. The presence of a central glucose molecule and multiple hydroxyl moieties (Table 1), together with its molecular weight, enables tannic acid to interact strongly with protein via multiple noncovalent interactions. As can be seen in Figure 4A,B, the visualized results confirmed the formation of multiple H-bonds between the phenolic OH groups of tannic acid with the protein functional groups (-N- or -O-); thus, tannic acid molecules served as a molecular bridge to connect protein chains into bundles. A total of 15 hydrogen bonds within a cut-off of four angstroms were observed via the interaction of tannic acid with canola proteins. The most active canola protein residues involved in this interaction via hydrogen bonding through the participation of oxygen atoms of the tannic acid in the interactions are summarized in Table S1. Recently, the cross-link strategy based on the formation of an H-bond has been widely used to design high-performance plastics with remarkable mechanical performance. Hence, the formation of multiple H-bonds between proteins and cross-linkers can considerably improve the mechanical and physical performance of bioplastics without sacrificing the extensibility and toughness because of their directionality, versatility, and reversibility [56]. In addition, a set of hydrophobic and π-stacking interactions within the specified cut-off were found, which often play important roles in protein–ligand interaction. Such interactions are favorable as they exhibit multiple functionalities, such as mechanical tenability and thermal stability. Additionally, when tannic acid is docked with soybean proteins in its most favorable binding cavity, there are nine amino acid residues interacting by hydrogen bonds, seven hydrophobic interactions, and one salt bridge formed by the interaction between positively and negatively charged amino acid side chains within 4 Å. Genipin was also observed to interact with canola and soybean proteins (−45.79 kcal/mol and −46.99 kcal/mol, respectively), exhibiting a strong interaction with a set of amino acid residues. Genipin cross-links canola proteins through strong hydrogen bonding of N-H-O and H-N-H of the cysteine, arginine, and asparagine groups. The complex is also stabilized by a set of hydrophobic interactions associated with genipin and the surrounding protein residues. Moreover, glutaraldehyde and maleic anhydride were found to stabilize the protein by hydrogen bonds along with hydrophobic interactions (Table S1). The contribution of such interactions to the fabrication of thermostable proteins has been previously demonstrated and shown to be crucial for its unusual high thermal stability [57].

In this section, the compatibility ranking between two polymers are described and proposed. Polymer blending approach offer an accessible route for the development of novel, high-performance polymeric materials. It can produce materials with better properties than its individual counterpart [58]. However, this section aims to predict the compatibility of the blend based on the strength of the polar group’s interaction between biopolymers and their role in the miscibility of the resulting biopolymer blend. The study is based on assessing the binding scores and the formation and number of hydrogen bonds to attain the miscibility of the blend. The role of hydrogen bonds as stabilizing interactions became an interesting subject of biopolymer blend research and development. It has been demonstrated that the formation of intermolecular hydrogen bonds between polymer blends not only enhances polymer blend miscibility or compatibility but also effectively promotes different properties of the polymer [59].

**Figure 5:**
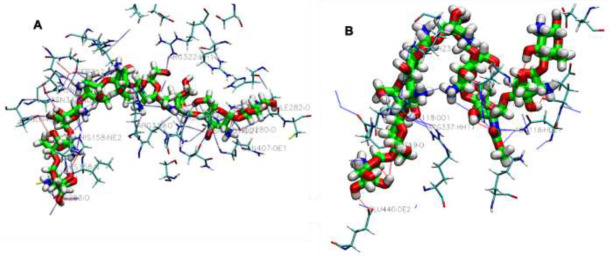
Three-dimensional view of protein–chitosan interactions: Canola protein (**A**), and Soybean protein (**B**).

In these docking studies, different types of polymers as additives were docked into protein structures and ranked by the calculated affinity. As can be seen, in Table 4, variations in the binding scores have been obtained across the whole range of polymers investigated. In this scene, the binding affinities of the seven polymer ligands in this class were docked with the two proteins. As shown in Table 4, the results of MM-GBSA on the polymer-binding system fall within a rather broad range, from −28.73 to −85.09 kcal/mol, show variation in interactions among these polymers. Visualization of the complexes (Table S1) indicated that the formation of different noncovalent interactions presents between the polar groups of polymers used as ligands and charged groups on the protein surface. These interactions very often lead to an increase in the compatibility and stability of the blends, which are essential to performing desired functions required for the intended application. In a molecular simulation, direct polar interactions are considered by specific terms such as hydrogen bonding, which play an important role in the stability of protein–ligand and polymer–polymer complexes and are required for most high-affinity ligands. Among the set of the covered polymers, chitosan (Figure 5A,B, Table 4) was predicted to be the most compatible with the investigated protein. Accordingly, the presence of primary and secondary reactive hydroxyl groups -OH groups and NH2 groups in the chitosan generates strong hydrogen bonds and salt bridges between the two polymers. Therefore, these newly generated hydrogen bonds are the key to the interaction between the protein and chitosan, favoring the compatibility of the system. The results are in good agreement with the experimental results for blending chitosan with plant canola and soybean proteins. Experimental observations guided by advanced analytical techniques revealed that the addition of chitosan to canola proteins enhances the mechanical properties of the canola protein films, from 15.4% to 25.0%. Hydrogen bonding was found to be the main force that mediates the interaction between blended polymers, thus contributing to good compatibility. The same study revealed that the complexation of chitosan and protein leads to polymer morphological changes [60]. In another study, the chitosan–soybean protein blends displayed strong interactions mediated by hydrogen bonding, leading to significantly improved mechanical properties in terms of tensile strength and elongation at the break of the bioplastic by 118.78% and 74.93%, respectively. Moreover, the resulting bioplastic shows a higher water contact angle and degradation temperature than that of pure soybean protein bioplastic [61]. Another study investigated the compatibility between soybean protein and agar, and their finding indicated that the formation of new hydrogen bonds between soybean protein and agar blends enhanced the compatibility and subsequently improved the mechanical properties [62]. As can be seen, our molecular docking results showed good agreement with the experimental results; thus, they can be used as a straightforward tool in the prediction of the compatibility and phase behavior of biopolymer blends.

## 4. Assessment of Docking Accuracy

In order to evaluate the quality of the results and the performance of the tools used, binding scores were evaluated using AutoDock Vina and Molecular Operating Environment docking protocol and compared to that of MM-GBSA prime. Up to 600 poses were explored in each compound with an induced fit of the protein and ranked all docked poses. The top 10 poses reported in each compound relied on London delta G as the S score.

Binding scores of the best conformer of each additive are given in Table 2, Table 3 and Table 4. It is important to emphasize that AutoDock Vina and MOE calculate docking scores with different search algorithms. A comparison of the affinity order from each approach revealed the same general trend, implying a common ground between AutoDock Vina and MOE since the ranking of the additives in both docking programs appeared to be the same. The majority of the poses predicted by MOE were located in close proximity to the site observed in AutoDock Vina. In comparison to the MM-GBSA results, a noteworthy feature of the results was that MM-GBSA had an almost similar trend but with a noticeable difference between the binding scores and the values of MM-GBSA. These findings prompted us to theoretically introduce MM-GBSA as a reliable indicator for ranking bioplastics additives. 

However, to validate our docking predictions, a statistical correlation was built between the docking scores of AutoDock Vina and MOE as well as between MM-GBSA and AutoDock Vina to see whether the binding scores predicted by AutoDock Vina, and MOE can correlate with MM-GBSA scores to correctly rank the binding affinities of the studied additives. The calculated binding affinity between plasticizers and cross-linkers with studied proteins from the AutoDock Vina and MOE correlated very well (correlation coefficients (r) of plasticizers = 0.9339 and 0.8457, correlation coefficient (r) of cross-linkers = 0.9301 and 0.9055, correlation coefficient (r) of blends 0.8787 and 0.9379 for canola and soybean, respectively). While it is clear that MM-GBSA showed stronger interaction than those predicted by docking scores, the finding showed that the AutoDock Vina and MOE results of plasticization and cross-linkers exhibited good rank correlation with the MM-GBSA results (correlation coefficient (r) = 0.9472, and 0.9197 for canola and soybean protein plasticization and 0.9465 and 0.9322 for canola and soybean protein cross-linking). Thus, the prediction performance of these methods was statistically identical and correlated strongly. However, the correlation coefficient of the AutoDock Vina results with MM-GBSA for polymer blending system of canola and soybean, are (r) 0.2339 and 0.2135, was not as satisfactory as that of low molecular weight additives such as plasticizers and cross-linkers. Thus, the rankings of large ligands with many rotatable bonds need to be confirmed experimentally.

In addition, a receiver operating characteristic curve (ROC) and area under the curve (AUC) were used in this study to obtain more accurate virtual screening results. The model’s performance of the AutoDock Vina method in attributing the best scores was confirmed by distinguishing false positives from true positives. Twenty decoy compounds were generated for phthalate and citric acid using the DUD-E platform. Then, the additives and decoy compounds were docked by the AutoDock Vina methodology, and the area under the ROC curve (AUC) was calculated for the two complexes. As illustrated in Figure S1, the docking results of the two additives (phthalate and citric acid) showed that the active molecules and inactive molecules could be properly docked into the active cavity. The AUC values of the phthalate–canola protein complex and phthalate–soybean protein complex was 0.7189 and 0.805, and those of the citric acid–canola protein complex and soybean protein complex were 0.8223 and 0.9611, indicating a good performance respectively. As can be seen from Figure S1, perfect performance is one that hugs along the outer left and top of the chart. Therefore, the ROC and AUC showed good results, confirming that the additives were properly docked into the active cavity using the AutoDock Vina methodology. It should be noted that the AUC value ranges from 0–1.0; a perfect performance will have an AUC of 1, whereas values of ≤0.5 imply a perfectly random process, while an AUC of 0 indicates a severe failure on the modeling side. At 0.728 to 0.93, our model’s AUC implies good performance. For the visualization, protein–additives complexes were visualized and double-checked using the Pymol, Discovery Studio, and VMD. Only confirmed and shared interactions between the three tools were considered in this study.

## 5. Conclusions

In the present study, we evaluated the performance of MM-GBSA, AutoDock Vina, and MOE to predict how plant canola and soybean proteins interact with additives. The binding poses and affinity of each investigated additive with the proteins were analyzed, and the preferred binding sites were determined. Binding scores were mainly used to rank twenty-one of the most widely used additives as ligands in protein bioplastic. 

Based on the results, it can be concluded that the investigated additives displayed a wide range of binding strengths, binding sites, and the number of formed hydrogen bonds, indicating variation in their strength of interactions. Therefore, an additive with a considerably stronger affinity is suggested to be selected as optimum for further development. Moreover, our results showed that MM-GBSA gave a better indication for protein–additives interactions and showed a good performance for additives with diverse molecular sizes and functional groups. Thus, our assessment of MM-GBSA in additives ranking is believed to show the best overall performance. Most notably, the interactions of plant protein–additives are very sensitive to the molecular size of the used additive and the pocket size of the host protein, which is directly related to the characteristics of the binding interface. In addition, our results showed that the protein–additive complexes are stabilized mainly by hydrogen bonds and hydrophobic and electrostatic forces. A variation in the density of the formed hydrogen bonds among the additives was observed, but the density of hydrogen bonds does not seem to have an effect on the strength of the interaction; therefore, the role of hydrogen bonding in additive efficiency needs to be assessed by molecular dynamic simulation. Certainly, our docking simulation results are in good agreement with the experimental measurements present in the literature. Therefore, the proposed method can be confidently used to predict the compatibility, efficiency, and volatility of the additives intended to be incorporated into the protein for the purpose of bioplastics preparation.

In summary, the molecular docking approach can serve as a fast and powerful tool in bioplastic additive ranking. This method allows for characterizing the compatibility and competition between protein and additives and between additives without the necessity of more sophisticated methods. Furthermore, implementation of such tools can help scientists to shorten the cycle of bioplastic development and thus make the process more cost-effective, obtain the hidden relationship between different variables, guide the chemical synthesis route, understand the mechanism of action, and identify and discover new potential additives with a significant reduction in cost and time. However, there is no doubt that the feasibility of molecular docking in predicting protein–additive interactions need to be experimentally examined. Obviously, comprehensive investigation behind every observation and optimizing the optimal computational protocol and the parameter in a single study is a non-simple task that requires more investigation, which could increase the accuracy and reliability of the results. 

## Figures and Tables

**Table 1:** The chemical structure and molecular weight of the compounds evaluated.

No.	Additive	PubChem CID	Molecular Weight g/mol	2D Structure
1	Formamide	713	45.041	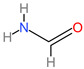
2	Ethylene glycol	174	62.07	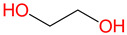
3	Urea	1176	60.056	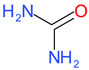
4	Glycerol	753	92.09	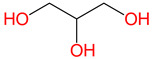
5	Triethanolamine	7618	149.19	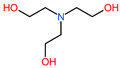
6	Sorbitol	5780	182.17	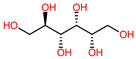
7	Phthalate	181977	164.11	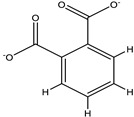
8	Glyoxal	7860	58.04	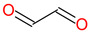
9	glutaraldehyde	3485	100.12	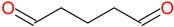
10	Maleic anhydride	7923	98.06	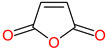
11	Succinic anhydride	7922	100.07	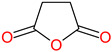
12	Citric acid	311	192.12	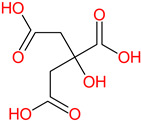
13	Genipin	442424	226.23	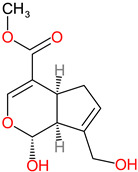
14	Tannic acid	16129778	1701.2	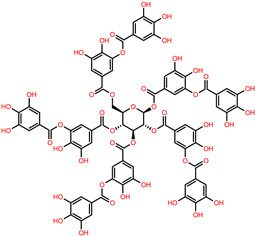
15	Cellulose	14055602	370.35	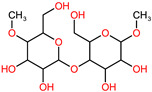
16	Starch	51003661	342.3	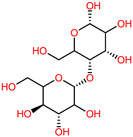
17	Agar	71571511	336.33	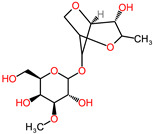
18	Kefiran	90908346	344.31	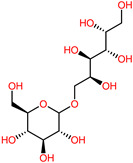
19	Lignin	73555271	1513.6	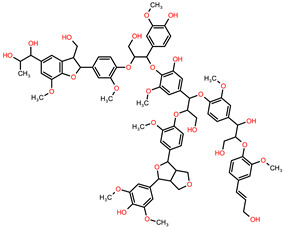
20	Dextran	4125253	504.4	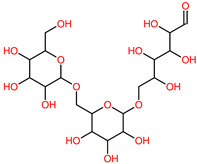
21	Chitosan	71853	1526.5	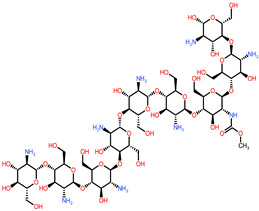

**Table 2:** Binding energies of protein–plasticizer interactions.

Plasticizer	Canola Protein			Soybean		
	Vina (kcal/mol)	MOE (kcal/mol)	MM-GPSA kcal/mol	Vina (kcal/mol)	MOE (kcal/mol)	MM-GPSA (kcal/mol)
Formamide	−2.7	−3.252	−14.85	−2.7	−3.408	−7.67
Urea	−3.5	−3.323	−15.44	−3.6	−3.874	−17.93
Ethylene glycol	−3.8	−3.830	−18.70	−3.8	−4,129	−13.29
Glycerol	−4.2	−4.698	−23.27	−3.9	−4.666	−35.58
Triethanolamine	−4.4	−5.564	−27.17	−3.9	−5.815	−36.15
Sorbitol	−4.9	−5.727	−28.86	−4.5	−6.313	−45.90
Phthalate	−5.4	−5.906	−30.05	−5.5	−6.325	−60.87

**Table 3:** Binding energies of protein–cross-linker interactions.

Cross-Linkers	Canola Protein			Soybean		
	Vina (kcal/mol)	MOE (kcal/mol)	MM-GPSA kcal/mol	Vina (kcal/mol)	MOE (kcal/mol)	MM-GPSA (kcal/mol)
Glyoxal	−3.1	−3.261	−11.00	−3.5	−3.874	−22.09
Glutaraldehyde	−3.3	−3.917	−13.84	−3.5	−4.355	−23.80
Maleic anhydride	−5.2	−4.049	−17.87	−4.4	−4.674	−25.22
Succinic anhydride	−5.2	−4.837	−19.44	−4.5	−5.383	−27.25
Citric acid	−5.9	−5.874	−28.90	−5.7	−6.029	−32.09
Genipin	−6.9	−9.639	−45.79	−5.8	−6.643	−46.99
Tannic acid	−9.1	−15.542	−52.21	−7.1	−12.125	−61.10

**Table 4:** Binding energies of protein–binder interactions.

Polymers	Canola Protein			Soybean		
	Vina (kcal/mol)	MOE (kcal/mol)	MM-GPSA kcal/mol	Vina (kcal/mol)	MOE (kcal/mol)	MM-GPSA (kcal/mol)
Cellulose	−6.1	−7.148	−62.65	−5.7	−5.941	− 49.15
Starch	−6.4	−7.340	−70.97	−5.8	−6.561	−76.67
Agar	−7.3	−7.542	−52.69	−6.1	−6.963	−44.82
Kefiran	−7.5	−7.782	−28.73	−6.2	−7.126	−35.43
Lignin	−8.9	−8.754	−52.08	−6.8	−8.235	−61.66
Dextran	−9.9	−8.935	−40.81	−7.9	−10.902	−56.53
Chitosan	−10.7	−15.325	−85.09	−10.8	−11.999	−61.87

## Data Availability

The data presented in this study are available on request from the corresponding author.

## Supplementary Materials

The following supporting information can be downloaded at: https://www.mdpi.com/article/10.3390/polym14173690/s1, Figure S1: ROC curves for docked canola- phthalate (A), soybean- phthalate (B), soybean-citric Acid (C) and canola-citric Acid (D) by AutoDuck Vina; Table S1: Protein-additive interaction profile; Table S2: Predicted binding sites of 11S globulin plant protein obtained by the different docking routines.
