# Supplementary material for: Assessing Molecular Docking Tools to Guide the Design of Polymeric Materials Formulations: A Case Study of Canola and Soybean Protein
Source: Polymers (Basel). 2022 Sep 5;14(17):3690. doi: 10.3390/polym14173690 (PMC9460131; doi:10.3390/polym14173690)
Supplement: Supplementary file 1 [file polymers-14-03690-s001.zip › polymers-1838029-supplementary.pdf]

Supplementary Information for

Assessing Molecular Docking Tools to Guide the Design of Polymeric Materials Formulations: A Case Study of Canola and Soybean Protein

Frage Abookleesh†, Farag E.S. Mosa‡, Khaled Barakat‡, Aman Ullah*†

‡Faculty of Pharmacy & Pharmaceutical Sciences, University of Alberta, Edmonton, AB, Canada T6G 2H5

†Department of Agricultural, Food and Nutritional Science, University of Alberta, Edmonton, AB, Canada T6G 2P5

*Corresponding author. Tel: +1 (780) 492-4845. E-mail: ullah2@ualberta.ca (Aman Ullah)

Table S1: Protein-additive interaction profile

Additives	Canola protein				Soybean protein				
	Residue	Distance (Å)	Bonds Involved	Interactions	Residue	Distance (Å)	Bonds Involved	Interactions	Interacting Atom/Group of Ligand
(1) Water	ASN 341C	1.99	O-H-O	HB	SER 63 B	2.10	O-H-O	HB	Oxygen
	ASN 341C	2.60	O-H-O	HB	SER 63 B	2.54	O-H-O	HB	Oxygen
	GLY383 C	2.14	O-H-O	HB	GLY 128 B	2.59	O-H-O	HB	Oxygen
	PHE 384 C	2.97	O-H-O	HB	VAL 129 B	2.58	O-H-O	HB	Oxygen
Formamide	ASN 311D	2.55	O-H-O	HB	ASN 325 A	2.10	O-H-O	HB	Oxygen
	SER 324D	2.32	O-H-O	HB	LYS 330 A	2.75	N-H-O	HB	Oxygen/Nitrogen
	ALA 325D	2.22	O-H-O	HB	GLU 324 A	2.34	O-H-O	HB	Carbon
	THR 309D	2.55	O-H-O	HB	ASN 355 B	2.69	O-H-O	HB	Carbon
	ALA 406D	2.21	N-H-O	HB	THR 358 B	2.70	O-H-O	HB	Oxygen/Nitrogen
Ethylene glycol	CYS 40D	2.02	O-H-O	HB	CYS 327 A	2.07	O-H-O	HB	Oxygen
	CYS 40D	2.19	O-H-O	HB	GLU 323 A	272	O-H-O	HB	Oxygen
	ASN 345D	2.33	O-H-O	HB	LYS 113 A	2.50	O-H-O	HB	Oxygen
					ARG 115A	3.72	O-H-O	HB	Oxygen
Urea	PHE 232D	2.55	O-H-N	HB	PRO 86 A	2.35	N-H-O	HB	Nitrogen
	GLY 413E	3.27	N-H-O	HB	THR 88 A	2.75	N-H-O	HB	Nitrogen
	ARG 419E	2.93	N-H-O	HB	GLN 323A	2.25	O-H-O	HB	Oxygen
	ARG 439E	3.04	O-H-N	HB	GLU323 A	2.18	N-H-O	HB	Oxygen
	VAL 440E	2.85	O-H-N	HB	THR 328 A	2.00	N-H-O	HB	Nitrogen
	THR 440 E	2.45	O-H-N	HB	ASP 342 A	2.34	N-H-O	HB	Nitrogen
Glycerol	LEU 181 F	1.81	O-H-O	HB	ASP 342 A	2.52	O-H-O	HB	Oxygen
	LEU 181 F	2.06	O-H-O	HB	ARG 340 A	3.16	O-H-O	HB	Oxygen
	ASN 186 F	3.06	O-H-O	HB	ASP 121 A	2.11	O-H-O	HB	Oxygen
	ASN 186 F	2.12	O-H-O	HB	ASN 334 A	3.05	O-H-O	HB	Oxygen
					GLN 333A	2.16	O-H-O	HB	Oxygen

Triethanolamine	ASN186 B	2.76	O-H-O	HB	HIS 332 B	2.17	O-H-O	HB	Oxygen
	GLN187 B	2.35	O-H-O	HB	LEU 331 B	1.96	O-H-O	HB	Oxygen
	ARG 190 B	2.44	N-H-O	HB	LYS 113 B	2.28	O-H-O	HB	Oxygen
	ASN 345 C	2.47	O-H-O	HB	SER 339 A	2.17	O-H-O	HB	Oxygen
	CYS 40C	1.89	O-H-O	HB	ASP 342 A	2.73	O-H-O	HB	
	ALA 182 C	3.83	C-C	HP					Carbon
**Sorbitol**	ILE 208 F	2.64	O-H-O	HB	THR 358 A	2.12	O-H-O	HB	Oxygen
	GLU 209 F	2.19	O-H-O	HB	THR 328 B	1.82	O-H-O	HB	Oxygen
	ASP 32 F	2.35	O-H-O	HB	LYS 113 B	2.47	O-H-O	HB	Carbon
	HIS 34 B	2.15	O-H-O	HB	ARG 340 A	2.24	O-H-O	HB	Oxygen
	HIS 19 B	2.31	O-H-O	HB	ASN 334 A	2.30	O-H-O	HB	Oxygen
	TRP 207 B	2.47	O-H-O	HB	ASP 342 A	2.21	O-H-O	HB	Oxygen
	ARG 190 D	2.22	O-H-O	HB	LYS 353 A	3.48	C-C	HP	Carbon
					SER 352A	3.09	C-C	HP	Carbon
Phthalate	ASN 343D	2.03	O-H-O	HB	LEU 331 A	2.58	O-H-O	HB	Oxygen
	ASN 403D	2.77	O-H-O	HB	HIS 332 A	3.28	O-H-O	HB	Oxygen
	GLN 158F	3.88	O-H-O	HB	LYS 330A	3.72	C-C	HP	Oxygen/ Carbon
	GLN 407D	3.98	C-C	HP	THR 358 B	3.82	C-C	HP	aromatic
	HIS 158 F	3.88	C-aromatic	SB	LYS 113 A	3.57	C-aromatic	SB	Carboxylate
					ARG115 A	3.94	C-aromatic	SB	Carboxylate
					Lys 113 A	3.69	C-C ring	π-stacking	Aromatic
Glyoxal	SER 18 B	2.93	O-H-O	HB	THR 88 A	2.22	O-H-O	HB	Oxygen
	ASP 32 B	2.12	O-H-O	HB	LYS 113 A	2.10	O-H-O	HB	Oxygen
	GLN 213 F	2.81	O-H-O	HB					
Glutaraldehyde	HIS 158 B	2.19	O-H-O	HB	LYS 113 B	3.58	O-H-O	HB	Oxygen
	GLU 280 B	2.77	O-H-O	HB	ARG 115 B	3.34	O-H-O	HB	Oxygen
	VAL 80 B	3.69	C-C	HP	THR 88B	3.53	C-C	HP	Carbon
	HIS 158 B	3.68	C-C	HP	GLN 112 B	3.86		HP	Carbon

	VAL 161 B	3.66	C-C	HP	LYS 113 B	3.45	C-C	HP	Carbon
maleic anhydride	TYR 196 B	2.93	O-H-O	HB	LYS 113 A	2.92	O-H-O	HB	Oxygen
	TYR 196 B	3.10	O-H-O	HB	ARG 115 A	3.51	O-H-O	HB	Oxygen
	TYR 56 B	2.85	O-H-O	HB	GLU 323 A	3.25	O-H-O	HB	Oxygen
	VAL 46 B	3.75	C-aromatic	HP	THR 88A	3.41	C-aromatic	HP	aromatic
	VAL 46 B	3.72	C-aromatic	HP	THR 88A	3.72	C-aromatic	HP	aromatic
	PHE 61 B	3.70	C-aromatic	HP					
Citric Acid	LEU 188 B	3.35	O-H-O	HB	LYS 113 B	2.07	O-H-O	HB	Oxygen
	HIS 184 B	2.25	O-H-O	HB	GLU 333A	2.14	O-H-O	HB	Oxygen
	LUE 188 B	3.15	O-H-O	HB	ARG 340 A	2.25	O-H-O	HB	Oxygen
	ARG 190 B	2.44	O-H-O	HB	ASP 342 A	1.81	O-H-O	HB	Oxygen
	ARG 190 C	2.33	O-H-O	HB	ASN334 A	2.74	O-H-O	HB	Oxygen
Genipin	CYS 40 E	2.25	O-H-O	HB	ASN 334 A	3.07	O-H-O	HB	Oxygen
	ARG 190 D	2.40	O-H-O	HB	THR 353 A	1.74	O-H-O	HB	Oxygen
	ASN 345 E	2.77	O-H-O	HB	ARG 115 B	2.85	O-H-O	HB	Oxygen
	GLN 187 D	3.58	O-H-O	HB	LYS 330 B	3.71	C-C	HP	Carbon
	GLN 187 D	3.20	C-C	HP	ARG 340 A	3.52	C-C	HP	Carbon
	ALA 182 E	3.65	C-C	HP	THR 358 A	3.65	C-C	HP	Carbon
Tannic Acid	GLN 1 C	3.13	O-H-O	HB	LYS 75 A	3.38	O-H-O	HB	Oxygen
	ASP 10 C	3.39	O-H-O	HB	HIS 116 B	2.79	O-H-O	HB	Oxygen
	ASP 10 F	1.88	O-H-O	HB	LYS 330 A	1.98	O-H-O	HB	Oxygen
	GLN 11C	2.34	O-H-O	HB	HIS 332 A	3.44	O-H-O	HB	Oxygen
	GLN 11 F	2.30	O-H-O	HB	ARG 350 B	2.89	O-H-O	HB	Oxygen
	ALA 14 C	2.00	O-H-O	HB	GLN 333 A	2.25	O-H-O	HB	Oxygen
	ARG211 B	2.75	O-H-O	HB	ARG 337 A	2.51	O-H-O	HB	Oxygen
	GLN 214 E	2.14	O-H-O	HB	SER 339 B	2.31	O-H-O	HB	Oxygen
	LYS 217 E	2.99	O-H-O	HB	ASN 334 A	2.59	O-H-O	HB	Oxygen
	GLN 214 B	3.00	O-H-O	HB	VEN 117 B	3.28	O-H-O	HB	Oxygen
	ASN 221 E	2.47	O-H-O	HB	ARG 340 B	3.20	O-H-O	HB	Oxygen
	GLN 245 E	2.16	O-H-O	HB	ARG 350 B	2.89	O-H-O	HB	Oxygen
	ARG 366 F	2.48	O-H-O	HB	ASN 118 A	3.54	C-H	HP	Oxygen

	ASN 363 F	3.38	O-H-O	HB	LYS 250 A	3.85	C-H	HP	Carbon
	ASP 10 C	3.80	-C-H-	HP	ARG 337 B	3.78	C-H	HP	Carbon
	GLN 11 C	3.28	-C-H-	HP	ARG 340 B	3.58	SB		Carbon
	ASN 13 C	3.89	-C-H-	HP	LYS 250 A	3.76	C-C rimg	π-stacking	Oxygen/aromatic
	ARG 366 F	3.61	-O-N-	SB	ARG 340 A	3.86	C-C rimg	π-stacking	Oxygen/aromatic
	GLU 6 E	2.96	O-H-O	HB	ASN 118 A	1.92	N-H-O	HB	Oxygen
	ALA 182 D	1.92	O-H-O	HB	LYS 330 A	2.92	N-H-O	HB	Oxygen
	ALA 182 D	3.16	O-H-O	HB	HIS 332 A	3.04	O-H-O	HB	Oxygen
	LEU 188 E	2.40	O-H-O	HB	ASN 334 A	3.09	N-H-O	HB	Oxygen
Cellulose	LEU 188 E	3.43	O-H-O	HB	ARG 337 A	2.56	N-H-O	HB	Oxygen
	ASP 189 D	3.61	O-H-O	HB	ARG 337 B	2.99	N-H-O	HB	Oxygen
	ARG 190 D	2.39	N-H-O	HB	SER 339 A	2.72	O-H-O	HB	Oxygen
	ARG 190 D	2.11	N-H-O	HB	ARG 340 A	2.1	N-H-O	HB	Oxygen
	ARG 190 E	2.43	N-H-O	HB	ARG 340 B	2.14	N-H-O	HB	Oxygen
	ARG 322 E	3.21	N-H-O	HB	ARG 340 B	2.13	N-H-O	HB	Oxygen
	PHE 3E	3.38	O-N	HP					Carbon
	GLU 6 E	3.87	O-N	HP					Carbon
starch	LYS 113 B	2.71	N-H-O	HB	ASP 342 A	1.92	O-H-O	HB	Oxygen
	ARG 115 B	2.59	N-H-O	HB	LYS 113 B	1.87	N-H-O	HB	Oxygen
	THR 328 B	1.95	O-H-O	HB	SER 339 A	1.97	N-H-O	HB	Oxygen
	LYS 330 B	1.96	N-H-O	HB	HIS 332 B	3.16	N-H-O	HB	Oxygen
	LEU 331 B	3.09	N-H-O	HB	ARG 115 B	3.69	O-H-O	HB	Oxygen
	HIS 323 B	1.90	O-H-O	HB	ARG 340 A	2.00	O-H-O	HB	Oxygen
	GLU 333 A	1.82	N-H-O	HB	LYS 330 B	2.38	O-H-O	HB	Oxygen
	ASN 334 A	2.41	O-H-O	HB	ASN 334 A	2.03	N-H-O	HB	Oxygen
	SER 339 A	2.73	O-H-O	HB	THR 328 B	2.71	O-H-O	HB	Oxygen
	ARG 340 A	1.86	N-H-O	HB	ARG 111 A	2.46	O-H-O	HB	Oxygen
	ASP 342 A	2.96	O-H-O	HB	GLN 88A	3.82	O-H-O	HB	Oxygen
	THR 353 A	3.30	N-H-O	HB	PHE 209 A	3.71	C-C	π-stacking	Oxygen
	LYS 113 B	3.89	N-O	SB	LYS 307 A	3.55		SB	Carboxylate
	ARG 340A	3.95	N-O	SB	LYS 87 A	3.74		SB	Carboxylate

Agar	ALA 182 B	2.21	O-H-O	HB	ASN 118B	2.03	O-H-O	HB	Oxygen
	HIS 184 B	3.73	N-H-O	HB	HIS 332 B	2.17	N-H-O	HB	Oxygen
	ARG 190 B	2.31	N-H-O	HB	LYS 330 B	2.38	N-H-O	HB	Oxygen
	ARG 190 B	3.30	N-H-O	HB	ARG 337 B	1.99	N-H-O	HB	Oxygen/Carbon
	ARG 190 C	2.72	N-H-O	HB	ARG 337 B	3.22	O-H-O	HB	Oxygen
	ARG 322 B	2.33	N-H-O	HB	SER 339 A	2.06	O-H-O	HB	Oxygen
	HIS 184 B	3.82	C-N	HP	ARG 340 B	3.15	N-H-O	HB	Carbon
	SER 183 B	3.35	C-N	HP	ASN 334 B	3.67	C-C	HP	Carbon
	GLU 6 C	3.89	C-C	HP	ARG 340 A	3.99	O-N	SB	Oxygen
kefiran	GLU 6 D	2.61	O-H-O	HB	ASN 118 B	2.97	N-H-O	HB	Oxygen
	GLU 6 D	2.70	O-H-O	HB	ASN 118 B	3.13	O-H-O	HB	Oxygen
	GLU 6 D	2.97	O-H-O	HB	ASP 121 B	3.63	O-H-O	HB	Oxygen
	LEU 181 F	2.20	O-H-O	HB	ASP 121 A	3.57	O-H-O	HB	Oxygen
	ASN 186 F	3.01	N-H-O	HB	LYS 330 A	2.54	N-H-O	HB	Oxygen
	ASN 186 F	1.95	O-H-O	HB	LYS 330 B	2.08	N-H-O	HB	Oxygen
	GLN 187 F	2..05	O-H-O	HB	HIS 332 A	2.11	O-H-O	HB	Oxygen
	GLN 187 F	3.05	N-H-O	HB	HIS 332 B	3.19	O-H-O	HB	Oxygen
	LEU 188 F	2.34	O-H-O	HB	GLN 33A	2.21	O-H-O	HB	Oxygen
	ARG 190 F	2.35	N-H-O	HB	ASN 334 B	2.61	N-H-O	HB	Oxygen
	ASN 345 D	3.16	N-H-O	HB	ARG 337 A	2.60	N-H-O	HB	Oxygen
	ALA 182 D	3.98	C-C	HP	ARG 337 B	3.40	N-H-O	HB	Carbon/Oxygen
	ARG 190 F	3.78	C-C	HP	ARG 337 A	3.28	N-H-O	HB	Carbon/Oxygen
					ARG 340 A	2.30`	N-H-O	HB	Oxygen
					ARG 340 B	3.19	N-H-O	HB	Oxygen
					ARG 340 B	3.70	C-N	SB	Carbon
					ARG 337 A	3.90	C-N	SB	Carbon
lignin	SER 312 B	2.34	O-H-O	HB	ASN 118 A	3.18	N-H-O	HB	Oxygen
	ARG 319 A	2.46	N-H-O	HB	ASN 118 B	3.23	N-H-O	HB	Oxygen
	ARG 322 A	3.36	N-H-O	HB	ASP 121 B	2.17	O-H-O	HB	Oxygen
	ARG 322 B	3.00	N-H-O	HB	LYS 250 A	2.68	N-H-O	HB	Oxygen
	ARG 322C	3.14	N-H-O	HB	TRP 251 A	3.14	N-H-O	HB	Oxygen
	ARG 322 C	2.69	N-H-O	HB	LYS 330 A	2.57	N-H-O	HB	Oxygen
	ASN 343 C	2.25	N-H-O	HB	LYS 330 B	3.21	N-H-O	HB	Oxygen

	ASN 403 C	2.40	N-H-O	HB	HIS 332 B	3.00	N-H-O	HB	Oxygen
	ALA 404 A	2.14	N-H-O	HB	GLN 333 B	2.28	O-H-O	HB	Oxygen
	ASN 405 B	2.98	N-H-O	HB	ASN 334 B	1.90	N-H-O	HB	Oxygen
	ARG 319 B	3.45	O-C	HP	ARG 337 B	2.56	N-H-O	HB	Oxygen
	PHE 320 B	3.99	C-C	HP	SER 339 B	3.23	O-H-O	HB	Carbon/Oxygen
	ARE 322 A	3.95	C-C	HP	ARG 340 A	3.13	N-H-O	HB	Carbon/Oxygen
	ASN 405 B	3.78	O-C	HP	ARG 340 B	3.36	O-H-O	HB	Carbon/Oxygen
					GLN 440 A	2.39	O-H-O	HB	Carbon/Oxygen
					LYS 250 A	3.85	C-C	HP	Carbon
					ALA 336 B	3.74	C-C	HP	Carbon
					PRO 338 B	3.87	C-C	HP	Carbon
	GLU 6 E	2.67	O-H-O	HB	HIS 116 A	3.73	O-H-O	HB	Oxygen
	GLU 6 E	2.43	O-H-O	HB	ASN 118 A	2.07	O-H-O	HB	Oxygen
	ASN 186 D	3.62	O-H-O	HP	ASN 118 B	2.09	N-H-O	HB	Oxygen
	GLN 187 D	2.81	O-H-O	HP	ASP 121 B	3.03	O-H-O	HB	Oxygen
	GLN 187 D	2.72	N-H-O	HB	LYS 330 A	3.15	N-H-O	HB	Oxygen
	LEU 188 D	2.73	N-H-O	HB	LYS 330 B	3.1	N-H-O	HB	Oxygen
	ARG 190 D	3.07	N-H-O	HP	HIS 332 A	2.23	N-H-O	HB	Oxygen
Dextran	ARG 190 D	3.19	N-H-O	HP	HIS 332 B	2.26	N-H-O	HB	Oxygen
	ARG 322 E	2.82	N-H-O	HB	GLN 333 A	2.72	O-H-O	HB	Oxygen
	ASN 345 E	2.83	N-H-O	HB	GLN 333 B	2	O-H-O	HB	Oxygen
	THR 402 E	3.48	N-H-O	HB	ASN 334 A	1.95	N-H-O	HB	Oxygen
					ASN 334 B	2	N-H-O	HP	Oxygen
					ARG 337 A	1.96	N-H-O	HP	Oxygen
					ARG 337 B	2.85	N-H-O	HP	Oxygen
					SER 339 A	3.47	O-H-O	HB	Oxygen
					SER 339 B	2.27	O-H-O	HB	Oxygen
					ARG 340 A	2.58	N-H-O	HB	Oxygen
	ILE 282 C	2.44	O-H-O	HB	GLN 440 A	2.11	O-H-O	HB	Oxygen
	GLU 280 C	2.66	N-H-O	HB	GLN 119 A	2.07	O-H-O	HB	Oxygen
	GLN 407 A	2.48	N-H-O	HB	LYS 250 A	2.26	N-H-O	HB	Oxygen
	ASN 343 A	2.47	N-H-O	HB	ASN 118 A	2.53	N-H-O	HB	Oxygen
	ASN 403 A	2.23	N-H-O	HB	ASN 118 A	2.29	N-H-O	HB	Oxygen

Chitosan	ARG 319 A	3.43	N-H-O	HB	ASN 118 A	23.18	O-H-O	HB	Oxygen
	ASN 405 B	2.63	N-H-O	HB	ASN 118 B	2.89	N-H-O	HB	Oxygen
	ASN 343 B	3.25	N-H-O	HB	ARG 337 B	2.06	N-H-O	HB	Oxygen
	HIS 158 A	2.44	N-H-O	HB	ARG 337 B	2.02	N-H-O	HB	Oxygen
	GLN 407 B	2.48	N-H-O	HB	ARG 337 B	2.27	N-H-O	HB	Oxygen
	GLN 407 B	3.01	O-H-O	HB	GLY 74 A	2.87	O-H-O	HB	Oxygen
	HIS 184 A	3.37	N-H-O	HB	THR 395 A	3.65	O-H-O	HB	Oxygen
	ARG 322 A	3.45	N-H-O	HB	LYS 250 A	3.60	N-O	SB	Oxygen
	THR 402 A	2.99	O-H-O	HB					
	TYR 313 B	2.19	O-H-O	HB					

Table S2: Predicted binding sites of 11S globulin plant protein obtained by the different docking routines.

Plant protein	Docking tool	Ligand	Binding score Kcal/mol	Residue active sites	Ref
Soy glycinin A3B4 subunit	Vina	Glycerol	-4.3	TH487, GLN110, THR326, PHE8, LYS111	[55]
Soy glycinin A3B4 subunit	Vina	Sorbitol	-5.5	LYS11, LYS328, SER350, THR351, THR357 LEU352	[55]
Soy glycinin A3B4 subunit	SYBLY	C3G	NA	SER90, ASP282, THR82, GLU84, PRO86, GLN88, SER242, PRO243, GLU281 and GLU283	[45]
grape seed 11S globulins	Vina	mv3glc)	-7.6 /-8.1	GLN 235, ASN 227, ARG 299, GLY 267, ASN 219, ARG 2010, PRO 208	[56]
soy glycinin A3B4 subunit	Schrödinger Glide	EGCG	−9.663	GLU172, HIS173, VAL162, GLY202	[57]
soy glycinin A3B4 subunit	AutoDock4.2.6	EGCG	-9.21	Gln73, Glu119, Glu139, Tyr371, Tyr393, Arg418 and Glu440	[58]
soy glycinin A3B4 subunit	CHARMM force field	ECCG/ catechin		ARG 418, TYR371, GLU31, ARG22, LY49, TYR 293, ARG18, VAL 404, PRO382, LEU 473	[59]
11S seed globulin	AutoDock Tools-1.5.6	Curcumin	-5.85	ARG158,TYR161,GLN168,ARG158, ARG159, PHE160,TYR161, IS166, GLU167,GLN168, LN176, ASN188 and ASN189	[60]
		Quercetin	-5.66	ARG158, TYR161, GLN176ARG158, PHE160, TYR161, HIS166, GLU167, GLN168, GLN176, ASN188 and ASN189, ARG158, PHE160 and ASN188	[60]
		Resveratrol	5.64	TYR161, GLN181,PHE160, TYR161, HIS166, GLU167, GLN168, LEU171, GLY178, GLY179, GLN181 and ASN188	[60]
11S seed globulin	PatchDock algorithm	anthocyanins	−40.52	Ser151, Leu152, Glu153, Asn154, Gln155, Gly47, Asp148, Asn150, Ser151, Leu152, Arg337, Asn358, Ala359, Gln397, Thr417, Asn418, Asp419, Asn150, Leu152, Glu153, Asn154, Gln155, Trp335, Arg337, Asn358, Asn418, Asp419	[61]
11S seed globulin	CHARMm forcefield	proanthocyanidin	−50.56 kcal/mol	Leu 58, Pro 93, Glu 95, Leu 162, Trp 129, Val 82, Val 127, Pro 61, Arg 102, Arg 103, Arg 60, Ala 163, Glu 218, Gly 164, Arg 212, Gly 215, Asn 189, Leu 213, Phe 203, Phe 191	[62]

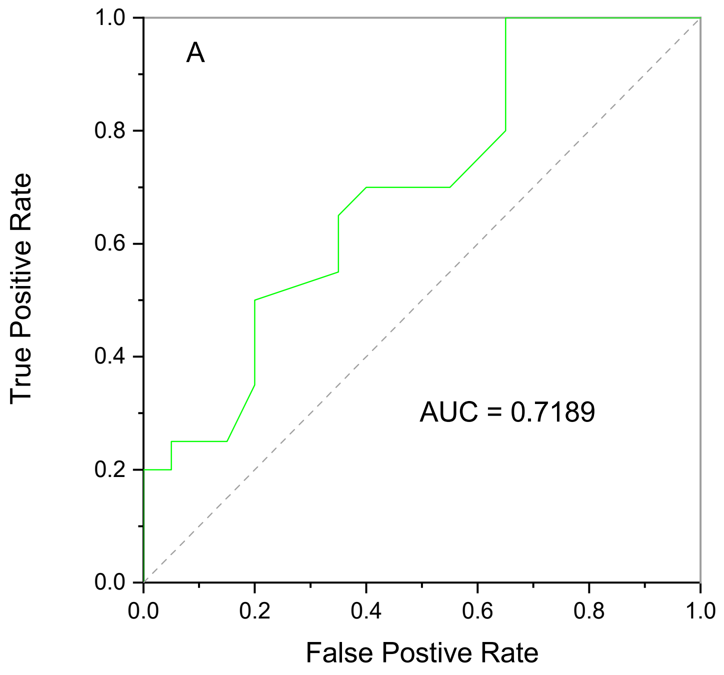

A
AUC = 0.7189
True Positive Rate
False Postive Rate
0.0
0.2
0.4
0.6
0.8
1.0

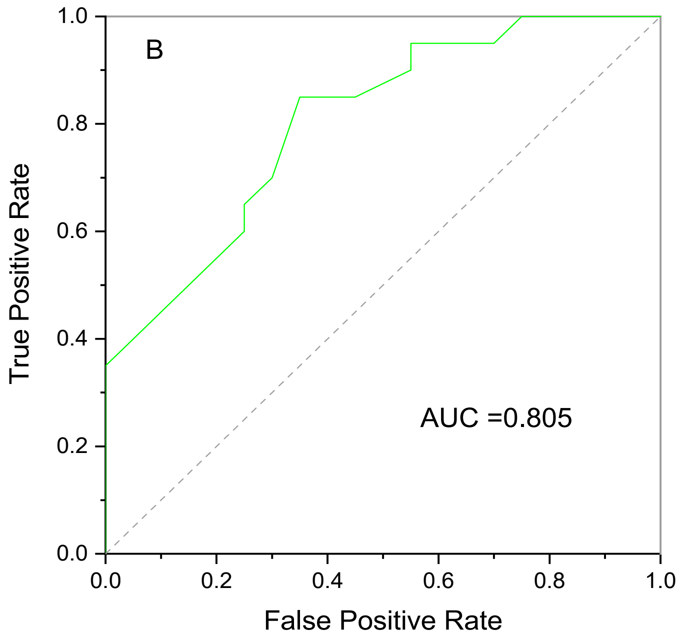
B
AUC =0.805
True Positive Rate
False Positive Rate
0.0
0.2
0.4
0.6
0.8
1.0

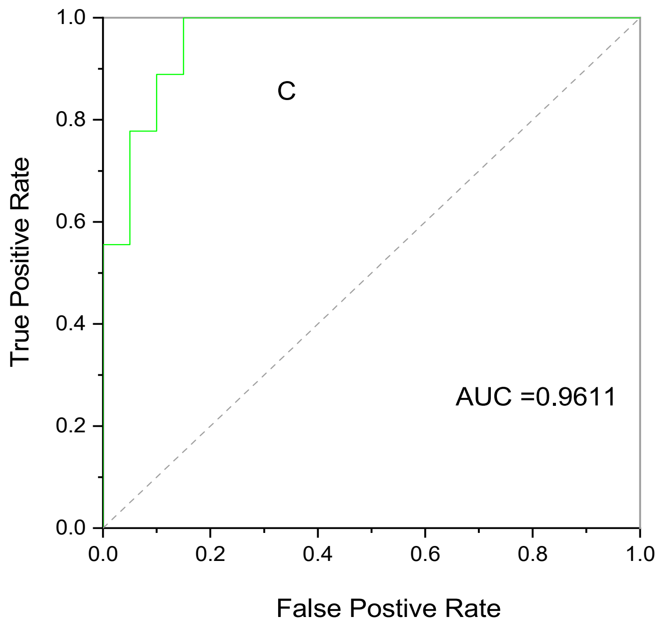
C
AUC =0.9611
True Positive Rate
False Postive Rate
0.0
0.2
0.4
0.6
0.8
1.0

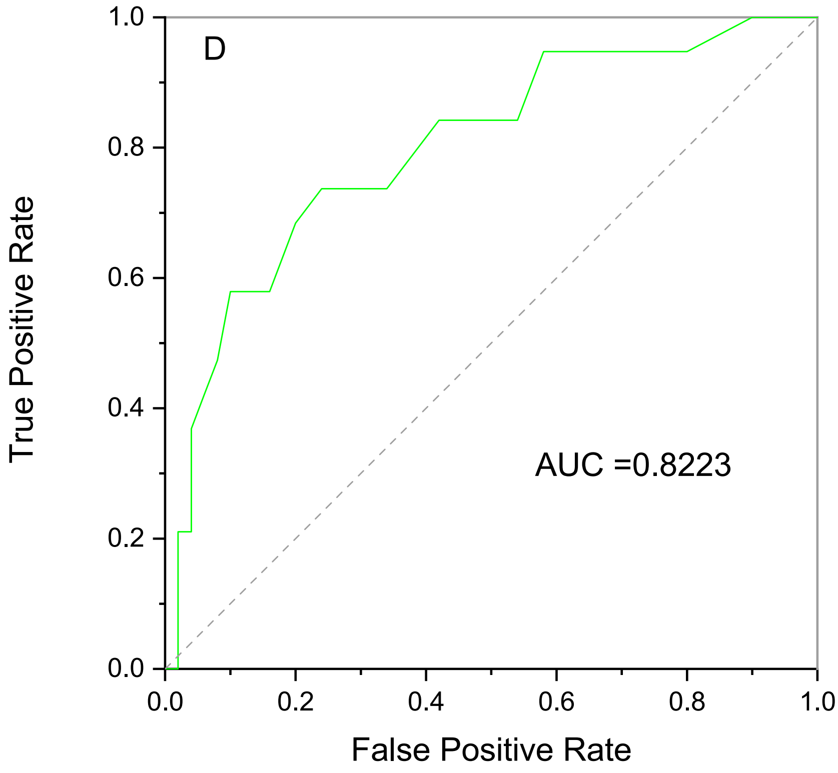

Figure S1: ROC curves for docked canola- phthalate (A), soybean- phthalate (B), soybean-citric Acid (C) and canola-citric Acid (D) by AutoDuck Vina
